# TAILORED INTERNET-BASED SEXUAL EDUCATION FOR OLDER ADULTS: NEEDS ASSESSMENT AND ALGORITHM DEVELOPMENT

**DOI:** 10.1093/geroni/igac059.2184

**Published:** 2022-12-20

**Authors:** Liza Berdychevsky, Galit Nimrod, Wendy Rogers

**Affiliations:** University of Illinois at Urbana-Champaign, Champaign, Illinois, United States; Ben-Gurion University of the Negev, Beer Sheva, HaDarom, Israel; University of Illinois Urbana Champaign, Champaign, Illinois, United States

## Abstract

Many older adults are sexually active, but ageism fuels the neglect of older adults' sexuality in research and sexual education. The purpose of this study was to (1) assess older adults’ willingness to adopt technology tools to receive innovative, tailored sexual health education via the internet, (2) investigate their sexual health needs, and (3) contribute to development of an assessment tool and tailoring algorithm. Tailoring is a precision approach that matches educational content to users’ needs instead of offering the same suboptimal one-size-fits-all content to everyone. We conducted an online survey using Qualtrics research panels of older adults (N = 836, with equal representation by gender and balanced representation in each 5-year cohort from age 60 to 80+). We found that 30% reported they were likely to try tailored internet-based education for older adults; an additional 34% were unsure. The results establish priority areas in sexual health needs, including behavioral patterns, knowledge gaps, health-related constraints, and attitudinal and communicational issues. These components provide the basis for the tailoring assessment questionnaire. Respondents also rated the personal relevance of 28 educational modules covering sex in later life (e.g., sexual ageism, sex as quality-of-life issue, sex-related communication, body image, health conditions, gender-specific issues, sexual attitudes, broadening sexual definitions and repertoires, sexual adaptations, inhibitions, sex aids/toys/robotics, medications/treatments, orgasm, self-stimulation/masturbation, dating, risk-taking, celibacy, LGBTQIA+, sexual rights, abuse). Statistical relationships between sexual health needs and personally relevant modules contribute to the development of a tailoring algorithm for creating educational bundles matched to older adults’ unique needs.

